# How to read neuron-dropping curves?

**DOI:** 10.3389/fnsys.2014.00102

**Published:** 2014-05-30

**Authors:** Mikhail A. Lebedev

**Affiliations:** Department of Neurobiology, Center for Neuroengineering, Duke UniversityDurham, NC, USA

**Keywords:** neuron-dropping curve, brain-machine interface, large-scale recording, neuroprosthetic device, neuronal noise, neuronal tuning, neuronal ensemble recordings

Methods for decoding information from neuronal signals (Kim et al., 2006; Quiroga and Panzeri, 2009) have attracted much interest in recent years, in a large part due to the rapid development of brain-machine interfaces (BMIs) (Lebedev and Nicolelis, 2006; Lebedev, 2014). BMIs strive to restore brain function to disabled patients or even augment function to healthy individuals by establishing direct communications between the brain and external devices, such as computers and robotic limbs. Accordingly, BMI decoders convert neuronal activity into font characters (Birbaumer et al., 1999), position and velocity of a robot (Carmena et al., 2003; Velliste et al., 2008), sound (Guenther et al., 2009), etc.

Large-scale recordings, i.e., simultaneous recordings from as many neurons and as many brain areas as possible, have been suggested as a fundamental approach to improve BMI decoding (Chapin, 2004; Nicolelis and Lebedev, 2009; Schwarz et al., 2014). To this end, the dependency of decoding accuracy on the number of recorded neurons is often quantified as a neuronal dropping curve (NDC) (Wessberg et al., 2000). The term “neuron dropping” refers to the procedure, where neurons are randomly removed from the sample until there is only one neuron left. In this analysis, large neuronal populations usually outperform small ones, the result that accords with the theories of distributed neural processing (Rumelhart et al., 1986).

In addition to BMIs based on large-scale recordings, several studies have adapted an alternative approach, where a BMI is driven by a small neuronal population or even a single neuron that plastically adapts to improve BMI performance (Ganguly and Carmena, 2009; Moritz and Fetz, 2011). In such BMIs, a small number of neurons serve as a final common path (Sherrington, 1906) to which inputs from a vast brain network converge.

While the utility of large-scale BMIs vs. small-scale BMIs has not been thoroughly investigated, several studies reported that information transfer by BMIs starts to saturate after the population size reaches approximately 50 neurons (Sanchez et al., 2004; Batista et al., 2008; Cunningham et al., 2008; Tehovnik et al., 2013). In one paper, this result was interpreted as mass effect principle, i.e., stabilization of BMI performance after neuronal sample reaches a critical mass (Nicolelis and Lebedev, 2009). However, another recent paper claimed that large-scale recordings cannot improve BMI performance because of the saturation (Tehovnik et al., 2013). This controversy prompted me to clarify here what NDCs show, whether or not they saturate, and how they can be applied to analyze BMI performance.

## Simulating NDCs in MATLAB

**Figure 1:**
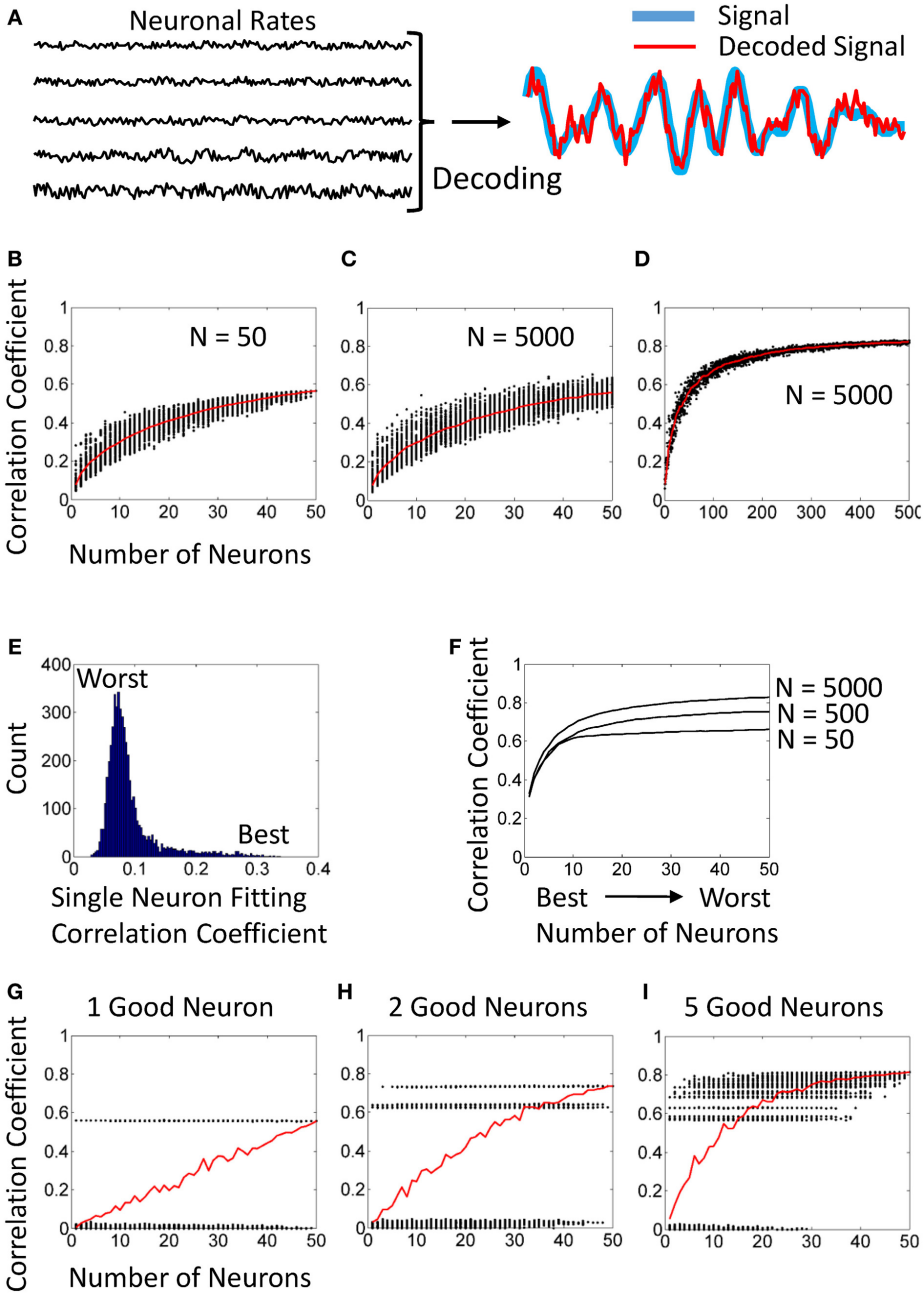
**Simulated NDCs. (A)** Simulated neuronal rates (left) and their utilization for decoding (right). **(B)** An NDC generated from a population of 50 neurons. Each dot corresponds to a randomly drawn neuronal subpopulation. **(C)** An NDC generated from a population of 5000 neurons with the same statistical distributions of tuning characteristics as for 50 neurons in panel **(B)**. **(D)** The same analysis as in **(C)** for subpopulation sizes from 1 to 500. **(E)** Distribution of single-neuron correlation coefficients for fitting. Computed for the same population of 5000 neurons as in panels **(C,D)**. **(F)** Ranked NDCs for populations of 50, 500, and 5000 neurons. The best tuned neuron was included first, followed by second best, etc. **(G)** An NDC for a population of 50 neurons, where one neuron is tuned, and 49 produce noise. **(H)** An NDC for 2 good and 48 noisy neurons. **(I)** An NDC for 5 good and 45 noisy neurons.

To produce illustrations of NDC characteristics (Figure 1), I used a simple simulation in MATLAB. The simulation was conducted over a time interval, *Interval*, which consisted of two parts: *Training_interval* used to train the decoder and *Test_interval* used to conduct decoding:

Interval=[Training Test];

To mimic typical BMI conditions, both intervals were set to 10 min, while the sampling rate was 10 Hz.

Neuronal rates were simulated as mixtures of signal and noise:

$$\begin{array}{l} Rate(:,\;i)=Signal\\ \;+\;Asynchronous\_noise\;(:,\;i)\\ \;+\;C{\left(i\right)}^{*}Common\_noise; \end{array}$$

where *Rate* is discharge rate, *i* is index that enumerates neurons, *Asynchronous_noise* is noise unique to each neuron, *Common_noise* is noise common to all neurons, and *C(i)* is the amplitude of common noise for each individual neuron. *Signal* and *Common_noise* were simulated as mixtures of sinusoids. *Asynchronous_noise* was produced by MATLAB random number generator.

Simulated neuronal rates are shown in the left part of Figure 1A. For the purpose of this simulation, it was not important to reproduce single-unit activity in all details (e.g., mimic refractory periods). In facts, many BMIs discard these details and utilize multi-units (Chestek et al., 2011).

I used multiple linear regression as the decoder (Wessberg et al., 2000; Carmena et al., 2003; Lebedev et al., 2005). This decoder was trained using MATLAB *regress* function:

$$\begin{array}{l} \text{B}=regress\;(Signal\;(Training),\\ \;[Rate(Training,:)\;ones\\ \;(size\;(Training^{\prime }))]); \end{array}$$

where B returned regression weights (one weight per neuron).

The decoded signal was calculated as:

$$\begin{array}{l} Decoded\_signal=[Rate\;ones\\ \;{\left(size(Rate,\;1)\right)]}^{*}B; \end{array}$$

Note that the values of *Decoded_signal* for the training interval represent fitting (i.e., decoding is produced from the data that were used to train the decoder), whereas the values for the test interval correspond to decoding *per se* because they are derived from new data. Figure 1A shows example traces of the signal (blue curve) and decoded signal (red curve).

Decoding accuracy was evaluated as correlation coefficient, *R*, for the test interval:

$$\begin{array}{l} R=corrcoef\;(Decoded\_signal\;(Test),\\ \;signal\;(Test);R=R(1,\;2); \end{array}$$

## Do NDCs saturate?

BMI decoding improves with the size of neuronal sample for a simple reason: noise is reduced and signal is enhanced by ensemble averaging. For the asynchronous noise, overall noise reduction is approximately proportional to the square root of the sample size. Therefore, adding more and more neurons to a population should result in a continuous improvement of decoding. Why, then, do NDCs saturate?

Figure 1B illustrates a typical NDC with a saturation effect. Here, different-size subpopulations were randomly drawn from a fixed neuronal sample (*N* = 50). The scatterplot represents decoding accuracy for each subpopulation as a black dot, and the red curve is the average NDC. Note that the scatter becomes narrow when subpopulation size approaches 50, which seems to indicate that decoding accuracy has saturated.

However, the analysis of Figure 1B is flawed because the data points to the right part of the curve are not statistically representative. Indeed, when the subpopulation size is close to 50, repeated draws return mostly the same neurons. For example, in any two subpopulations of size 49, 48 neurons would be identical. Therefore, the NDC of Figure 1B converges to the value that depicts the performance of a fixed neuronal sample of *N* = 50 rather than representing random draws of such sample.

A different pattern is revealed when neuronal subpopulations are drawn from a much larger neuronal sample. In Figure 1C, subpopulations (1 to 50 neurons) were drawn from a fixed sample of 5000 neurons. This NDC does not saturate. In particular, the scatter of dots does not narrow down as dramatically as in Figure 1B, and correctly reflects the standard deviation for different subpopulation sizes.

Furthermore, subpopulation sizes from 1 to 500 are explored in Figure 1D. Here the original sample of 5000 neurons is the same as in Figure 1C. Clearly, this NDC indicates continuous improvement in decoding beyond 50 neurons.

In conclusion, NDCs lose statistical validity when subpopulation size gets comparable with the fixed population from which it is drawn. This may produce an illusion of saturation.

## Some neurons are more important

NDCs are usually computed as mean values for randomly drawn neuronal subpopulations. This representation masks the fact that individual neurons contribute unevenly to the decoding. There are leaders that represent parameters of interest particularly well, and there are noisy neurons with very small contributions to the decoding. Decoding usually improves if only the good neurons are utilized, and the noisy ones are discarded (Sanchez et al., 2004; Westwick et al., 2006).

Contribution of individual neurons to decoding (also called importance or sensitivity) can be estimated by running the decoder separately for each neuron. Here, depending on the analysis, individual correlation coefficients can measured for the training interval (i.e., fitting) or for the test interval (i.e., decoding). These two metrics are usually very similar. Figure 1E shows a distribution of correlation coefficients for fitting for 5000 individual neurons. Notice a distribution “tail” that corresponds to particularly good neurons.

After individual performance is evaluated, neurons can be ranked by their contributions to decoding. Furthermore, NDC subpopulations can be constructed starting with the best neuron, then adding the second and so on (Sanchez et al., 2004; Lebedev et al., 2008). Examples of such rank-ordered NDCs are shown in Figure 1D. Here, the analysis was performed for populations of 50, 500, and 5000 neurons. Notice that in each of these cases, the highest ranked 30–50 neurons performed practically as well, as the entire population. Still, the draws from 5000 neurons always outperformed 500 neurons, and 500 neurons outperformed 50 neurons; simply because better subpopulations could be picked when a large neuronal sample was available.

Notably, a selection of informative neurons for one behavioral parameter (e.g., hand coordinate) can be different from the selection for another parameter (e.g., gripping force or leg coordinate). Therefore, recordings from large neuronal populations become particularly important when several parameters need to be decoded simultaneously (Fitzsimmons et al., 2009).

Currently, little is known about types of neurons in cortical microcircuits (Casanova, 2013; Opris, 2013) that could be more useful for BMIs. It seems reasonable to assume that output neurons of such microcircuits could provide high quality BMI signals, but this issue needs more investigation. It would be of interest to record from an entire microcircuit, e.g., from a single cortical column, to reconstruct information processing and representation using BMI methods.

## Average NDCs may be misleading

Although average NDCs usually indicate a gradual improvement in decoding accuracy when more and more neurons are added, this representation may be misleading as it conceals the fact that there are only a few informative neurons in the population.

Figure 1G illustrates a population of 50 neurons with only one informative neuron and 49 neurons generating noise. Even in this extreme case, an average NDC indicates a gradual improvement. This is because the probability of the single good neuron to be present in a subpopulation increases with the subpopulation size. Similar gradually rising NDCs can be obtained with 2 (Figure 1H) and 5 (Figure 1I) good neurons.

These examples show that for the analysis to be complete, an average NDC should be supplemented by a ranked NDC (e.g., Figure 1F) and a plot of individual contributions of different neurons (Figure 1E).

## Common noise

Unlike asynchronous noise, common noise does not attenuate when firing rates of many neurons are averaged. Correlated variability in neuronal firing has been described in many cortical areas (Abbott and Dayan, 1999; Hansen et al., 2012; Opris et al., 2012). Whereas such correlations have important functions, they may be detrimental to BMI decoding if they are unrelated to the parameter being decoded. Interestingly, transition to online BMI control is accompanied by an increase in correlated variability (Nicolelis and Lebedev, 2009; Ifft et al., 2013).

Decoding in the presence of common noise can be improved by recording from multiple brain areas (Lebedev and Nicolelis, 2006; Nicolelis and Lebedev, 2009) because inter-area correlations are weaker than intra-area correlations (Ifft et al., 2013). Curiously, adding neurons that are poorly tuned to a parameter of interest, but have a considerable common noise, can improve the decoding. Indeed, the decoder could use such neurons to recognize common noise and to subtract it from the contribution of well-tuned neurons.

Overall, although handling common noise is not as straightforward as handling asynchronous noise, using large populations of neurons is beneficial here, as well.

## Offline vs. online NDCs

NDCs are often used to support the claim that BMIs perform better when they utilize large neuronal ensembles (Nicolelis and Lebedev, 2009). However, in the majority of cases NDCs are calculated offline instead of testing neuronal ensembles of different size in real-time BMIs. To the best of my knowledge, ensembles of different size were tested online in just one study (Ganguly and Carmena, 2009), where removal of neurons from an initial population of 15 neurons deteriorated monkey performance in a reaching task controlled through a BMI. Apparently, more studies of this kind will be needed in the future to clarify this issue in more detail.

An additional caveat of NDC analysis is related to its application to the data obtained during real-time BMI control. Although it might be tempting to apply a decoder or a tuning curve analysis (Ganguly and Carmena, 2009) to BMI control data, these considerations can easily get circular because the signal (e.g., cursor position) in such datasets has been already generated from neuronal activity by a decoding algorithm. To cope with this problem, NDCs could be calculated for a parameter that was not included in the decoding algorithm, for example target position (Ifft et al., 2013).

## Conclusion

Although NDCs often appear to saturate after neuronal population size reaches a critical value, a more careful consideration indicates that this effect may be an artifact of the overall limited neuronal sample. Large-scale neuronal recordings appear to be a realistic way to attain accuracy and versatility of BMIs.

### Conflict of interest statement

The author declares that the research was conducted in the absence of any commercial or financial relationships that could be construed as a potential conflict of interest.
